# Non-Uniform Distribution of Cryoprotecting Agents in Rice Culture Cells Measured by CARS Microscopy

**DOI:** 10.3390/plants10030589

**Published:** 2021-03-21

**Authors:** Fionna M. D. Samuels, Dominik G. Stich, Remi Bonnart, Gayle M. Volk, Nancy E. Levinger

**Affiliations:** 1 Department of Chemistry, Colorado State University, Fort Collins, CO 80523, USA; 2 Advanced Light Microscopy Core, NeuroTechnology Center, University of Colorado School of Medicine, Anschutz Medical Campus, Aurora, CO 80045, USA; DOMINIK.STICH@CUANSCHUTZ.EDU; 3 USDA-ARS National Laboratory for Genetic Resources Preservation, 1111 S. Mason St., Fort Collins, CO 80521, USA; remi.bonnart@usda.gov (R.B.); gayle.volk@usda.gov (G.M.V.); 4 Department of Electrical and Computer Engineering, Colorado State University, Fort Collins, CO 80523, USA

**Keywords:** cryopreservation, cryoprotectant distribution, raman microscopy

## Abstract

Cryoprotectants allow cells to be frozen in liquid nitrogen and cryopreserved for years by minimizing the damage that occurs in cooling and warming processes. Unfortunately, how the specific cryoprotectants keep the cells viable through the cryopreservation process is not entirely evident. This contributes to the arduous process of optimizing cryoprotectant formulations for each new cell line or species that is conserved. Coherent anti-Stokes Raman scattering microscopy facilitates the visualization of deuterated cryoprotectants within living cells. Using this technique, we directly imaged the location of fully deuterated dimethyl sulfoxide (d_6_-DMSO), the deuterated form of a commonly used cryoprotectant, DMSO, within rice suspension cells. This work showed that d_6_-DMSO does not uniformly distribute throughout the cells, rather it enters the cell and sequesters within organelles, changing our understanding of how DMSO concentration varies within the cellular compartments. Variations in cryoprotectant concentration within different cells and tissues will likely lead to differing protection from liquid nitrogen exposure. Expanding this work to include different cryoprotectants and mixtures of cryoprotectants is vital to create a robust understanding of how the distributions of these molecules change when different cryoprotectants are used.

## 1. Introduction

Preserving cells and tissues for later use is vital in fields from human in vitro fertilization and organ transfers to the preservation of agricultural crops or animals and endangered plant and animal species [1,2,3]. The importance of plant conservation is recognized by The Convention on Biological Diversity (2002), which had targets focused on conserving 75% of threatened plant species ex situ as well as the conservation of 70% of the genetic diversity of crops, wild relatives, and other economically relevant species by 2020 [4]. In the review of the program twelve years later it is clear that these goals were overly ambitious [5]. Falling short of the targets set in 2002 can be attributed to the recalcitrance of some species to traditional seed banking, such as those that have non-orthodox seeds or that are clonally maintained for which collections must be grown as plants in the field, greenhouse, or in vitro for preservation. These collections are particularly susceptible to environmental threats as well as pests, pathogens, and diseases. Having a secure backup of these collections is critical to their long-term sustainability. When possible, cryopreservation, or long-term storage in liquid nitrogen, provides a secure, efficient backup for plant genebank collections.

Cryopreservation has been utilized in plant genebanks for over thirty years. Cryopreservation methods have been developed to maximize the number of cells that survive storage in liquid nitrogen. These methods are effective at preserving biological materials, e.g., cells and tissues, stored in the liquid or vapor phase of liquid nitrogen [1,2,6]. Methods based on cryoprotectant solutions rely on bathing the materials in mixtures of molecules called cryoprotecting agents (CPAs) that protect cells and tissues from the mechanical and osmotic stresses associated with cooling and rewarming [6]. Examples in plant cryopreservation include Plant Vitrification Solution 2 (PVS2; 30% glycerol, 15% dimethyl sulfoxide, 15% ethylene glycol, 0.4 M sucrose; [7]) and Plant Vitrification Solution 3 (PVS3; 50% glycerol, 50% sucrose; [8]), both shown in the 1990s to be highly effective for cryopreserving cells and plant shoot tips. However, these methods are not universally protective, and each treatment must be optimized for each new plant species conserved. Optimizing these methods for individual plant species takes time and resources that may not be available for endangered species. Determining how they interact will establish how different CPAs protect specific parts of cells. Establishing this fundamental knowledge will guide practices and minimize the resources needed in the traditionally empirical approach to optimization. This work aimed to add to the growing body of literature concerned with CPA–cell interactions by demonstrating that coherent Raman microscopy enables direct visualization of deuterated CPAs within living plant cells.

## 2. Results and Discussion

**Figure 1:**
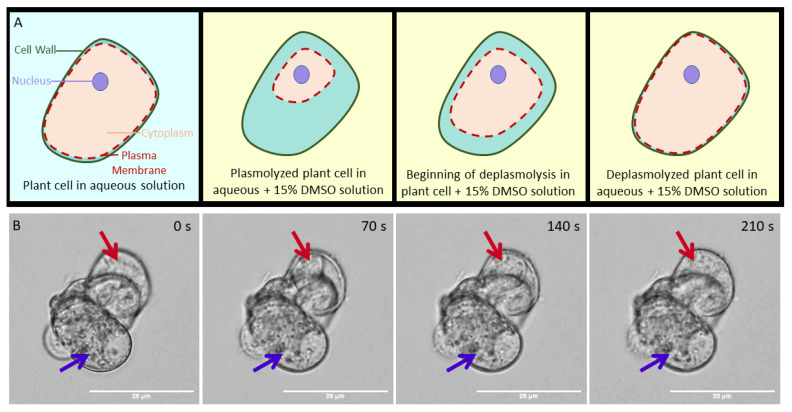
Panel A: Cartoon representation of a cell undergoing plasmolysis (the pulling away of the red dashed plasma membrane from the green cell wall) and deplasmolysis (the moving of the cell membrane back to cell wall) as it is exposed to a 15% DMSO in water solution (yellow background). Panel B: Brightfield microscopy images showing a small cluster of rice suspension cells 0, 70, 140, and 210 s after exposure to 15% aqueous DMSO. Red arrow: a cell that completely plasmolyzed after 70 s and deplasmolyzed after 210 s. Purple arrow: a cell that did not appear to respond to the 15% DMSO in water solution.

When plant cells are exposed to CPAs, they can go through a process of plasmolysis and deplasmolysis [9,10,11], as shown in Figure 1A. Plasmolysis is attributed to the change in osmotic pressure when a cell is exposed to a cryoprotectant solution, observable when the plasma membrane shrinks away from the cell wall. This is seen in Figure 1, Panel B, in the image acquired 70 s after exposure to 15% dimethyl sulfoxide (DMSO), a commonly used CPA in plant and animal cryopreservation. Following plasmolysis, the cell swells until the cell membrane reaches the cell wall in a process called deplasmolysis. Deplasmolysis is commonly attributed to CPAs entering the cell [9], as it is generally seen when cells are exposed to cell-permeating cryoprotectants, like DMSO. However, simple attribution of CPAs entering the cell to cause deplasmolysis does not explain why some cells, like the cell indicated by the purple arrow in Figure 1B, do not appear to respond to DMSO exposure with a plasmolysis/deplasmolysis cycle, while others, like the cell indicated by the red arrow, appear to respond as expected, plasmolyzing within 70 s of exposure and deplasmolyzing after 210 s of exposure, at room temperature. Both cells had movement within their cytoplasm, leading us to believe that they were both alive. This difference in response was observed in many different exposure experiments and with cells in clusters ranging from only a few cells to hundreds of cells (see Supplementary Information).

Prior to exposure, at 0 s, the two highlighted cells appear similar, both appearing populated with small organelles, which move within the cell. Thus, we might expect similar responses to the 15% DMSO solution. The question remains, did DMSO enter the unresponsive cell, or is it preferentially concentrated in the responsive cell? Although the macroscopic behavior of the cell is easily identifiable with bright field microscopy, the definitive localization of DMSO within these cells remains elusive. A primary objective of the current work was to directly observe the accumulation of DMSO within cells, as previous works have almost entirely relied on observing cellular responses to CPA exposure to understand the protective behavior of CPAs.

There has been some work to characterize the cellular responses to explore how CPAs work to protect or destroy cells [7,8,9,10,11,12,13,14,15,16]. Bright field microscopy techniques have shown how cells respond to CPA exposure in real-time [9]. Fluorescence microscopy has identified how fluorescently-labelled organelles move and change upon CPA exposure [12], as well as the histological changes that occur [11]. Electron microscopy has been used in conjunction with fluorescence microscopy to observe ultrastructural changes within cells that occur with CPA exposure and freezing [15,16]. Toxicity studies have demonstrated the applicability of standard CPA formulations to new cell lines [7,8], and various protein assays have determined DNA or RNA damage by CPA exposure [13,14]. All these studies inform how cells respond to and are damaged by CPA exposure, but they do not identify or determine CPA location or translocation within cells. Detailed information on CPA location and translocation within living cells, specifically which organelles are being most impacted by CPA exposure, will enable the development of highly robust and specific cryopreservation protocols that provide improved tolerance to cooling and low temperature storage.

Most CPA formulations are mixtures of small molecular components, commonly DMSO, glycerol, ethylene glycol, and sugars. These molecules cannot be directly imaged by bright field microscopy and attaching a dye molecule to any of these molecules dramatically changes their diffusion behavior, making bright field and fluorescence microscopies ill-suited for determining their exact location within cells. However, these small molecules can be imaged using vibrational microscopy, which enlists unique vibrations intrinsic to the molecules of interest to image the sample. Although IR microscopy may be used as a label-free imaging technique, its intrinsically low spatial resolution (due to long IR wavelengths) and water’s high IR absorptivity makes it challenging to image biological samples. Recent advances in IR microscopy that enlist both a mid-IR and ultraviolet laser can generate photoacoustic data with resolutions comparable to those collected with fluorescence microscopy [17]. Raman microscopy enables the imaging of live samples with wavelengths in the near-IR and visible region via the vibrations associated with specific molecules [18]. For example, the hydrocarbon in lipid molecules is highly effective for imaging cell membranes [18]. Although spontaneous Raman microscopy has been used to image live cells and tissues, it suffers from low sensitivity and contamination from endogenous sample fluorescence.

Various nonlinear coherent Raman scattering methods demonstrate dramatic enhancement in sensitivity over traditional Raman microscopy [19,20,21,22]. For example, coherent anti-Stokes Raman scattering (CARS), used in this work, boasts five to six orders of magnitude higher sensitivity to spontaneous Raman measurements [18,20]. Additionally, coherent Raman microscopy techniques avoid interference by endogenous fluorescence through optical filtering and also offer high spatial resolution, with optimal resolutions ≤300 nm, and high temporal resolution, <1 s, to measure location and translocation of molecules of interest [18,22]. These dramatic improvements enable real-time acquisition in live, biological samples [23].

All coherent Raman processes require two short laser pulses, usually in the picosecond range, aligned in space and time so that both beams simultaneously impinge on the sample. When the frequency difference between the pump and Stokes beams matches a molecular vibration in the sample, a CARS signal is generated (see SI) [20]. A CARS signal is quadratically proportional to the concentration of the molecule of interest [20], so CARS microscopy is most frequently used to image prevalent biological components in cells. Although Raman techniques have been used to image intra-cellular lipid responses to CPAs [24], freezing behavior of water in and around live cells exposed to CPAs [25,26,27], and bulk distributions of CPAs in frozen mixtures [28], they have yet to be applied to directly image CPAs distribution within living plant cells. In the work reported here, we used CARS microscopy to image deuterated dimethyl sulfoxide (d_6_-DMSO) interacting with live rice suspension cells. 

**Figure 2:**
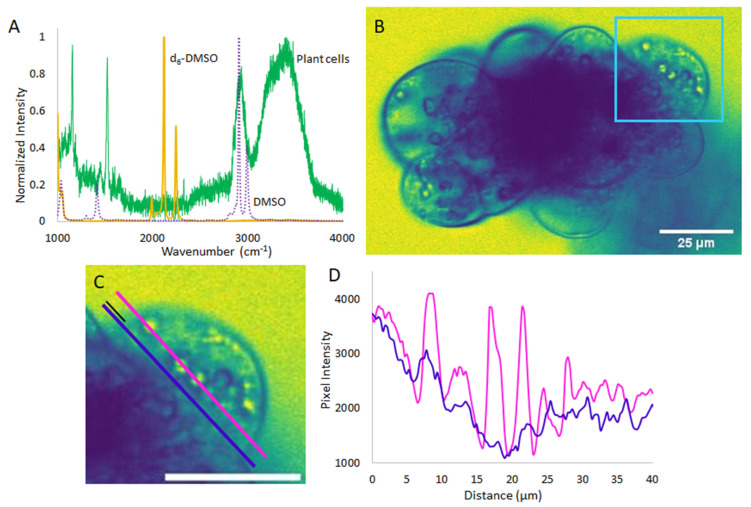
(**A**): Spontaneous Raman spectra of d_6_-DMSO (yellow), DMSO (purple, dashed), and the rice cells (green), showing the shift in DMSO vibrational frequency upon deuteration away from significant peaks in the cell spectrum. (**B**): Representative CARS microscopy image of rice cells imaged in resonance with the d_6_-DMSO stretching vibrational mode (yellow trace in **A**). Signal from d_6_-DMSO appears yellow while places blocking the d_6_-DMSO signal appear blue. (**C**): Expanded view of cell outlined in light blue in B. Parallel pink and dark blue line profiles bisect three organelles and the relatively uniform space away from organelles, respectively. Both lines start 5 µm outside of the cell, a distance shown with the small black line, and stretch, parallel, across approximately 40 µm. (**D**): Pixel intensity along pink and dark blue line profiles from C as a function of distance along the line. Scale bars in C and D are 25 µm. Images artificially colored with ImageJ LUT, mpl-viridis [29].

As most CPAs are organic molecules composed largely of carbon, hydrogen, and oxygen, their vibrational modes often fall in the same frequency range as the biological sample itself. In this work, we enabled selective CPA detection by deuteration. Figure 2A shows how deuteration in d_6_-DMSO shifts the C-H stretch of DMSO (~2900 cm^−1^) away from the broad C-H stretch region in the plant cells to the relatively quiet C-D stretch region of the spectrum (~2120 cm^-1^). Targeting the 2120 cm^−1^ stretch ensures that the CARS signal detected reflects d_6_-DMSO while cell features like the cell membranes and walls block that signal. The CARS image shown in Figure 2B was collected after the cell was exposed to d_6_-DMSO for approximately 3 min, which was the amount of time it took to prepare the sample and begin collecting data. The d_6_-DMSO signal appears as yellow, while blue features indicate places where no d_6_-DMSO is present or components of the cell or other cells block the d_6_-DMSO signal. Figure 2C shows an expanded view of the cell highlighted by the light blue square in Figure 2B. In this cell, the d_6_-DMSO signal appears to pool in subcellular organelles. That is, rather than remaining dispersed throughout the cytoplasm, the d_6_-DMSO preferentially accumulates in specific organelles. The blue outlines around these pools and in other parts of the image arise from areas where cellular components, such as lipid membranes and cell walls, block the d_6_-DMSO signal. Figure 2D contrasts the pixel intensity along the pink and dark blue lines drawn in Figure 2C. The pink line intercepts three obvious d_6_-DMSO-rich organelles while the blue line goes through a relatively uniform background part of the cell, contrasting the inside of the organelles to the surrounding cytoplasm. It is clear that there is a higher concentration of d_6_-DMSO inside those organelles than in the surrounding cytoplasm. This demonstrates that d_6_-DMSO is not uniform in the cell interior, preferentially pooling inside organelles within the cell. Other than a ~8% increase in mass, the properties of d_6_-DMSO differ little from those of H_6_-DMSO. Thus, we expect the same effect when cells are exposed to H_6_-DMSO, as the molar mass is not significantly changed by deuteration.

The CARS images collected of d_6_-DMSO indicate that DMSO is pooling in specific organelles within the live cells. On the basis of bright field microscopy experiments (shown in SI), we suspect that the organelles preferentially taking-up DMSO are amyloplasts and/or starch bodies. Additionally, there is evidence in the literature suggesting that DMSO interacts with glucose and amylose, both components of starch [30,31]. An increase in DMSO concentration in these organelles may increase the amount of protection afforded to the organelles as DMSO is known to disrupt the hydrogen bond network of water and support vitrification over ice crystallization. This result may also indicate that these organelles are at a higher risk of damage from DMSO toxicity, as DMSO has been shown to cause cell death [32] and, at high concentrations, is presumed to disrupt cell membranes [33,34]. Furthermore, the apparent sequestration of DMSO inside these organelles challenges the assumption of equal DMSO distribution throughout the cell. The preferential localization of the DMSO cryoprotectant has ramifications in all disciplines that use cryopreservation—an unequal distribution of cryoprotectants in cells and tissues have different implications depending on the specific system. In cells that do not contain these organelles, DMSO uptake may be more uniform or different organelles may be preferentially sequestering the cryoprotectant. Previous research on cellular responses to CPAs have made it obvious that assuming CPAs to work equally and effectively in all cell types is flawed [7,8], and this result may partially explain the differences in cellular response seen in brightfield studies like those in Figure 1.

## 3. Materials and Methods

### 3.1. Growth and Maintenance of Oryza sativa (Asian Rice) Cells

#### 3.1.1. Rewarming Cells and Initial Plating

Rice callus cells were acquired from the United States Department of Agriculture Agricultural Research Service (USDA-ARS) National Laboratory for Genetic Resources Preservation in Fort Collins, CO. The cell line was originally produced by G. Schaeffer, U.S. Dept. of Agriculture, Beltsville, MD in 1981 [35]. The rice cells used in this work were originally cryopreserved by Finkle and Ulrich in 1981 using PGD (10% *w*/*v* polyethylene glycol, 8% *w*/*v* glucose, and 10% *w*/*v* DMSO, [36]) and a slow-cool procedure. Rice callus cells (A7 line) were removed from liquid nitrogen and immediately warmed in a 40 °C water bath for 2 min until the solid cryoprotectant solution inside the vial was liquid. The cryoprotectant solution and cells were then diluted with 0.5 mL of wash solution made with 30 g L^−1^ sucrose (Alfa Aesar, Ward Hill, MA, USA) and Murashige and Skoog basal plant medium with vitamins (MS Media, M519; PhytoTechnology Laboratories, Lenexa, KS, USA) in distilled water at 22 °C and incubated for 10 min before 1 mL of wash solution was added at 22 °C. The solution was then allowed to sit for 10 min. The vial was then centrifuged for 1 min at 1000 rpm. The supernatant was removed with a pipette and 1 mL of wash solution was added to the remaining cells and they were incubated at 22 °C for 10 min. The liquid was removed from the vial with a pipette and the cells were scooped from the vial onto a sterile filter paper and blotted to remove excess liquid. The semi-dry cells were then plated onto solid modified MS Media (PT046; HiMedia Laboratories, Lincoln University, PA, USA) supplemented with 1 mg L^−1^ each of 2,4-dichlorophenoxyacetic acid (2,4-D, Sigma-Aldrich, St. Louis, MO, USA), indoleactetic acid (IAA, TCI America, Portland, OR, USA), and kinetin (TCI America), 146 mg L^−1^ glutamine (Acros Organics, Geel, Belgium), 30 g L^−1^ sucrose, and 8 g L^−1^ agar (BD Diagnostics, Franklin Lakes, NJ, USA) at pH 5.7. The plated cells were placed in a light-free container and were confirmed to be alive after growth was visible (approximately 3 weeks after plating).

#### 3.1.2. Cell Culture

The cells were grown as suspension cultures or as callus. For both suspension and plated cultures, modified MS medium (PT046) was supplemented with 1 mg L^−1^ each of 2,4-D, IAA and kinetin, 146 mg L^−1^ glutamine, 30 g L^−1^ sucrose, and 8 g L^−1^ agar (removed for suspension cells) at pH 5.7. After autoclaving, 10 mL of medium was placed in 6 cm diameter Petri dishes and allowed to set for 30 min before being stored in the refrigerator. Rice callus cells were transferred to new solid media every 4–6 weeks depending on growth of the callus. When they were transferred, growth appearing the lightest in color was selected from the callus with sterile tweezers and placed onto the new medium. The cells were grown at room temperature in a closed drawer, maintaining constant darkness, and used in experiments as necessary. Suspension cells were grown using the same formulation of modified Murashige and Skoog medium, without the addition of agar. To grow cells in suspension, approximately a gram of the lightest colored rice callus cells was removed from plates and placed in Erlenmeyer flasks with 50 mL liquid media. Cells were culture grown on a shaker continuously rotating at 140 rpm. The Erlenmeyer flasks with suspension cells were wrapped in aluminum foil to ensure the cells would be grown in the dark. Suspension medium was replaced every 1–2 weeks, and the cells were moved to new suspension cultures at that time, depending on growth. When creating a new culture, flasks were removed from the shaker and allowed to settle for about 10 min before excess media was removed with a pipette. Cells were removed from the flask, blotted on sterile filter paper to remove excess medium, and about 1 cm^3^ of cells was placed in a new flask.

### 3.2. Cryoprotectant Solutions

For bright field images, 15% (*w*/*v*) DMSO in distilled water was used. For all CPA exposures imaged with the CARS microscope, 15% (*w*/*v*) d_6_-DMSO (MilliporeSigma, St. Louis, MO, USA) in distilled water was used. This is the concentration of DMSO found in PVS2 (*5*) and was chosen for the broad applicability of DMSO to both animal and plant cryopreservation.

### 3.3. Cell Imaging

**Figure 3:**
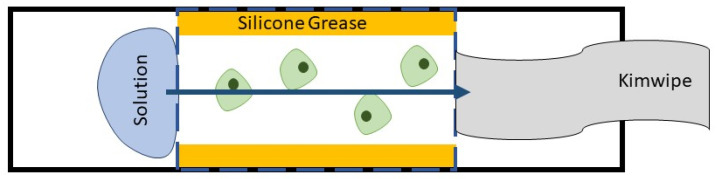
Rudimentary perfusion chamber showing plant cells in green, silicone grease in yellow, wicked solution in blue, and Kimwipe in grey on a white microscope slide (outlined in solid black). 25 × 50 mm cover slip placed on top of grease shown in blue dashed lines.

Bright field images were acquired using an Olympus IX73 fluorescence microscope (Olympus Corporation, Tokyo, Japan) in the Chemistry Department Cell Culture Facility at Colorado State University. A rudimentary perfusion chamber was developed to image cells as exposure to CPA solutions occurred. The rudimentary perfusion chambers were made using a microscope slide, cover slip, and silicone grease, as shown in Figure 3. The microscope slide was coated in 1% poly-L-lysine (Electron Microscopy Sciences, Hatfield, PA) to immobilize the rice cells while solutions flowed through the chamber. The slide was cleaned with methanol, then a large drop, enough to cover the entire area under the coverslip, of poly-L-lysine was placed on the slide and allowed to set for 24 h. After setting, the poly-L-lysine was rinsed away with distilled water and the slide was air dried and immediately used for imaging the cells. Both cells grown on plates and suspension cultures were used. Callus cells were suspended in a small amount of MS Media by placing a small amount of friable callus in a vial with 1 mL of media and vigorously shaking. Suspension cells were placed on microscope slides directly from the cellular suspension. After placing the cells on the microscope slide, a 25 × 50 mm cover slip was placed onto the slide, creating a wide channel through which CPA mixtures could be wicked. Approximately 0.5 mL of the 15% DMSO solution was placed on the edge of the coverslip while a Kimwipe was held to the other end. This created flow and images were acquired as the solution travelled across the perfusion chamber and the CPA solution was allowed to sit in the chamber. This was repeated five times with 15% DMSO in water and a total of 35 cells were clearly visible. Of these cells, 8 appeared dead, 22 cells (61%) had a visible response to the solution, and 3 cells (8%) had a full plasmolysis/deplasmolysis cycle. A visible response was considered a rapid shrinking and expansion where there was no visible plasmolysis. For more on the number of cells responding to CPA mixtures, see the SI.

See Supporting Information for a CARS microscopy instrument description.

### 3.4. IKI Staining

Cells were stained with an iodine solution prepared with 100 mL distilled water, 1 g of iodine chips (Fisher Scientific, Fair Lawn, NJ, USA), and 2 g KI (Fisher Scientific) for 1.5 to 5 minutes. After staining, the cells were rinsed with water before mounting and imaging.

## 4. Conclusions

Using CARS microscopy, we visualized sequestration of d_6_-DMSO in organelles within living rice suspension cells that were originally cryopreserved in 1981. After 37 years of cryostorage, the cells were thawed in 2018, and grew vigorous, friable calli. Both macroscopic signs of growth and generated autofluorescence within cells demonstrated the viability after rewarming and cryoprotectant exposure experiments. These cells serve as a model system for our first experiments demonstrating the value of direct visualization of cryoprotectants in living systems. Localization of cryoprotectants like DMSO in cell organelles may explain how these substances induce changes in cellular processes [14,32]. The nonuniform distribution of cryoprotectant in the cells implies that any assumption of an equal CPA dispersion within a cell, and consequently equal protection afforded by the CPA throughout the cell, is flawed. Continued investigation into the exact location of various CPAs within a broad range of living cells and tissues will likely illuminate why cellular response varies with CPA exposure, something applicable to both animal and plant cells. The establishment of cellular responses to CPA exposure has the potential to streamline development of cryopreservation protocols for newly endangered plant and animal species, allowing goals such as those set by The Convention on Biological Diversity to be more readily achievable, and enabling more species to be conserved as we face our current climate crisis.

## Data Availability

All data are available in the main text or the Supplementary Materials.

## Supplementary Materials

The following are available online at https://www.mdpi.com/2223-7747/10/3/589/s1, Figures S1–S11.
