# Supplementary material for: Non-Uniform Distribution of Cryoprotecting Agents in Rice Culture Cells Measured by CARS Microscopy
Source: Plants (Basel). 2021 Mar 21;10(3):589. doi: 10.3390/plants10030589 (PMC8003852; doi:10.3390/plants10030589)
Supplement: Supplementary file 1 [file plants-10-00589-s001.pdf]

Non-Uniform Distribution of Cryoprotecting Agents in Rice Culture Cells Measured by CARS Microscopy

Fionna M. D. Samuels, Dominik G. Stich, Remi Bonnart, Gayle M. Volk, Nancy E. Levinger
Correspondence to: fionna@colostate.edu and/or nancy.levinger@colostate.edu

Materials and Methods

CARS Microscopy Instrument Description

All coherent anti-Stokes Raman scattering (CARS) images were collected at the Advanced Light Microscopy Core (ALMC), at the UC Denver, Anschutz campus. The images were acquired with the help of the ALMC personnel, Dr. Dominik Stich and Greg Glazner. A detailed description of the microscope follows here.

The Advanced Light Microscopy Core coherent anti-Stokes Raman scattering (CARS) imaging system is composed of an APE (Angewandte Physik und Elektronik GmbH, Berlin) picoEMERALD optical parametric oscillator (OPO) and an inverted Olympus IX81 microscope (Olympus Corp., Tokyo) with an Olympus FV1000 scan head (Olympus Corp., Tokyo). The APE OPO incorporates a NKT aeroPulse PS10 fiber laser (NKT Photonics A/S, Denmark) that provides a pulse train centered at 1031 nm with an 80 MHz repetition rate. A portion of the 1031 nm beam is frequency doubled to 515.5 nm and used to generate the pump beam in the OPO while the remaining 1031 nm beam comprises the Stokes beam. The OPO offers a software-controlled tunable OPO signal and OPO idler output from 700 nm to 990 nm and 1080 nm to 1950 nm, respectively. The 1031 nm beam and the OPO signal are used for CARS imaging. The pulse widths for each beam is approximately 2 ps and the spectral bandwidth of the probed CARS signal is approximately 10 cm^{-1}. The pump and Stokes beam pulse trains are overlapped temporally and spatially before exiting the OPO aperture. The beams are reduced fourfold in diameter by a telescope before being coupled into the scan head of the microscope.

From the scan head, the beams enter the microscope stand through the rear port and are reflected toward the objective by a dichroic mirror in the stand turret (Chroma t750spxrxt, lot 217722). The beams are focused into the sample by an Olympus UPlanSApo 20X, NA 0.75 air objective.

The forward CARS signal is collected by a NA 0.55 air condenser (Olympus Corp., Tokyo) and detected by an Olympus non-descanned PMT Unit (FV10MP-BXTD, Olympus Corp., Tokyo). The laser beams are rejected by a shortpass filter (Thorlabs, FES750) and a bandpass filter (Chroma HG730/50M2P, lot 248096).

In general, a CARS Stokes beam fixed at 1031 nm and a CARS Pump/Probe beam tunable from 780 nm to 960 nm can generate CARS signal in a range from 720 cm^{-1} to 3120 cm^{-1}. With the described filters in the detection path, CARS signal can be detected in the range from 1775 cm^{-1} to 2240 cm^{-1}. In these experiments the CARS Pump/Probe beam was set to 845.0 nm to detect the CARS signal of the C-D stretch vibration in d_6-DMSO at 2120 cm^{-1}.

In addition to the CARS signal, two photon autofluorescence (TPA) from the pump beam was collected in the epi direction in a range from 400 nm to 600 nm with a second non-descanned detector Unit (Olympus FV10MP- *IXD2CH*, Chroma, e600sp-2p, lot 221876). The CARS system also offers the capability of CARS imaging in epi direction and SHG imaging in forward direction, as well as normal confocal scanning imaging, see Figure S1. These capabilities were not used in the reported experiments.

CARS and TPA images, z-stacks and time-lapses were recorded in the FV10-ASW 4.2 acquisition software (Olympus Corp., Tokyo).

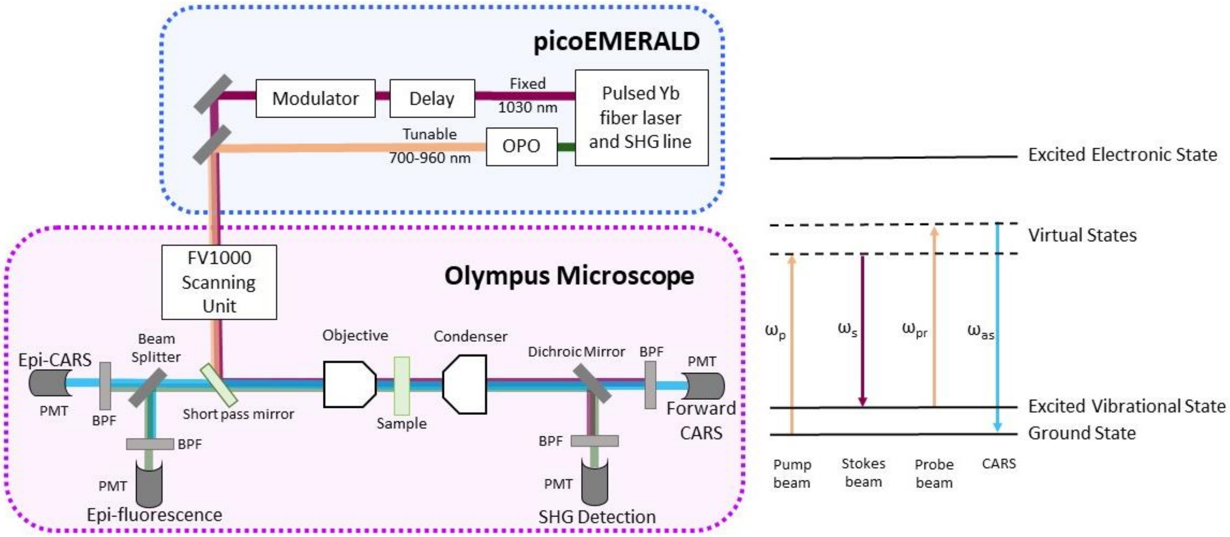

Figure S1. Simplified schematic of microscope set up and corresponding energy diagram of CARS process.

Supplementary Text and Figures

Bright Field Images

Figure S2 shows a sample of the IKI staining experiments carried out to help determine organelle identities IKI stains starch a dark brown, and it is clear in the figure that cells either contain a few starch bodies (presumably amyloplasts) or no starch bodies. These studies lead us to believe that the primary organelles that are similar in size to those seen sequestering d_6-DMSO in the CARS images collected are likely amyloplasts.

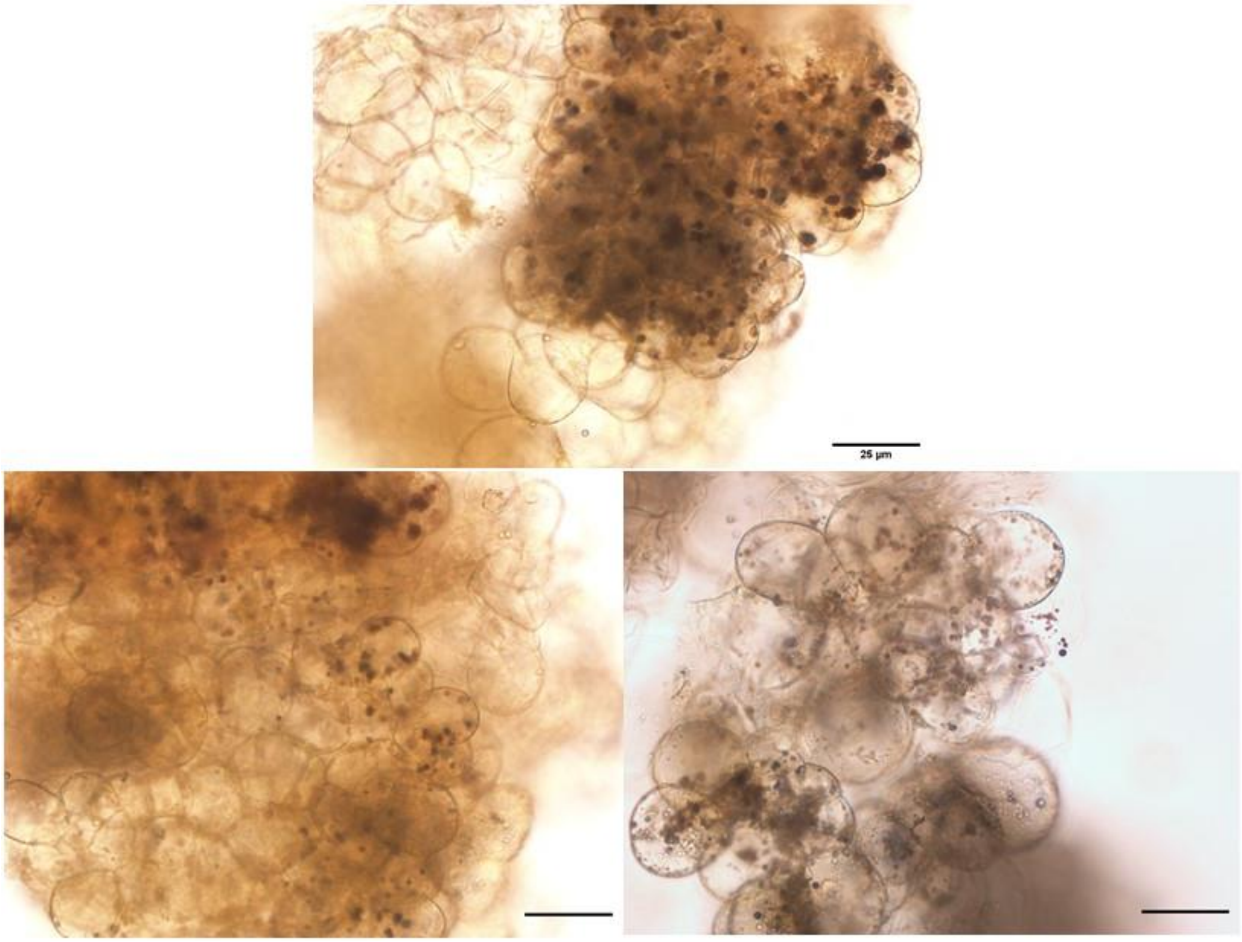

Figure S2. Starch containing organelles in rice callus cells stained with IKI for 5 minutes, 5 minutes and 1.5 minutes (top, bottom-left, bottom-right). All scale bars are 25 μm.

Figure S3 shows bright field images of callus cells being exposed to 10% DMSO. While this concentration is lower than that used in the primary CARS figure in the main text of the paper, the cellular response should be similar, if not the same, with a slightly higher DMSO concentration. The most likely difference in cellular response between a lower concentration will be to have a slightly smaller cellular response. In this case it is important to notice that the cell labelled with the pink arrow is one of the only cells obviously responding to the DMSO exposure at t = 2.2 min and that it appears to undergo plasmolysis twice. This is further evidence that directly imaging the exact location of cryoprotectants when they are interacting with plant callus cells is important and may inform further, unusual cell behavior.

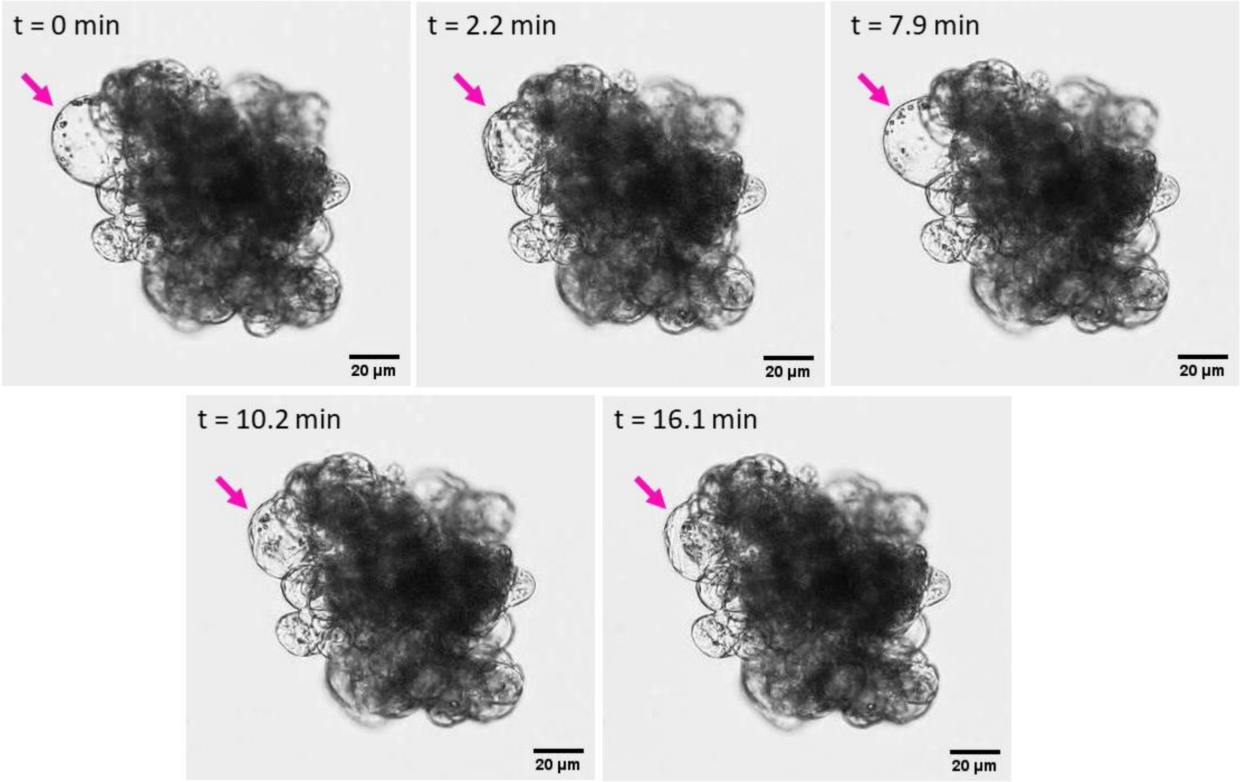

Figure S3. Bright field image of cells exposed to 10% DMSO in distilled water.

Figure S4 shows a bright field image of densely packed callus being exposed to 15% DMSO. Similar to the previous figure, only the cell labelled with the pink arrow appears to be responding as expected to the DMSO exposure, plasmolyzing and deplasmolyzing over the course of 11 minutes. Again, this further illustrates the need for a more foundational understanding of how exactly the cryoprotectants are being distributed through the callus.

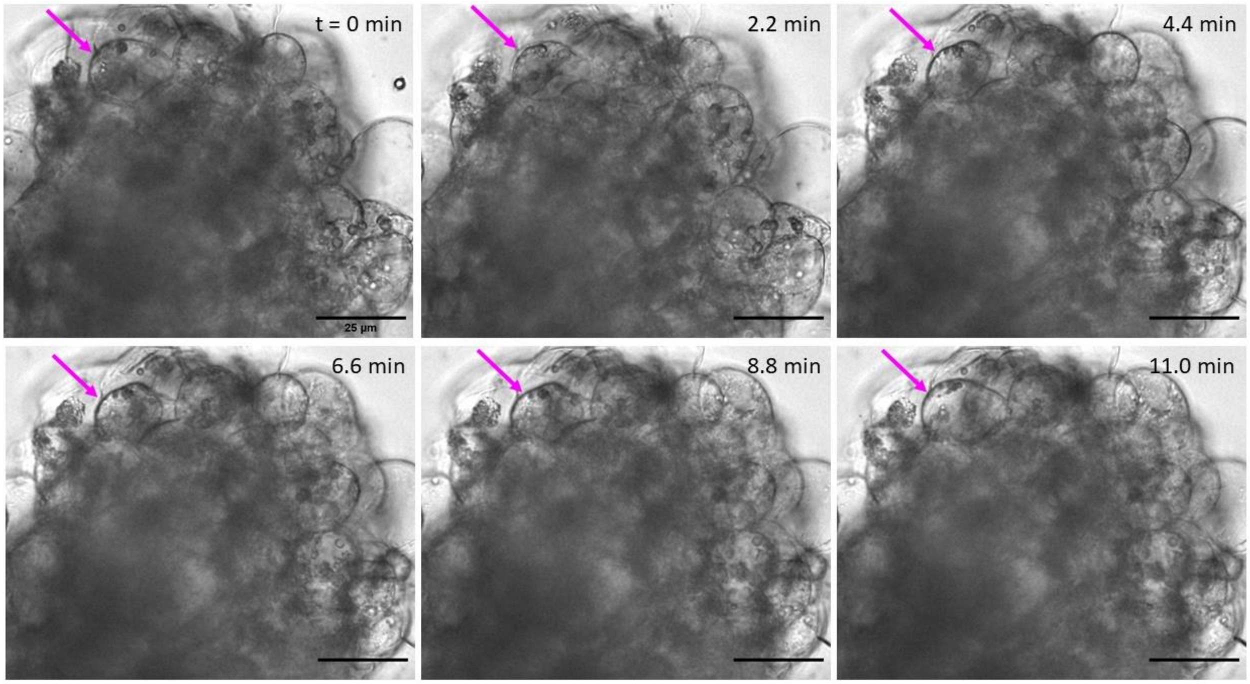

Figure S4. Bright field images of cells exposed to 15% DMSO in water. All scale bars are 25 μm.

Table S1: Counting data for different experiments showing the response of cells when exposed to different CPA solutions. Cells were considered responsive if there was a clearly shrunk and expanded immediately after exposure but did not plasmolyze. These cells were counted under "# Cells Responsive". For those experiments labelled 'na' in the "# Cells Responsive" column, image data were collected after initial exposure as there was not access to a perfusion chamber at the time. FOV is the field of view on the microscope.

CPA components	# Clear Cells in FOV	# Cells Responsive	# Cells that Plasmolyze/Deplasmolyze
10% DMSO 8% Glucose	19	na	2
10% DMSO 8% Glucose 10% EG	15	na	5
**Total**	34	na	7
10% DMSO in MS	10	9	1
10% DMSO in MS	19	5	1
10% DMSO in MS	9	9	0
10% DMSO in MS	21	6	0
**Total**	59	29	2

15% DMSO	4	4	1
15% DMSO	2	2	0
15% DMSO	8	0	0
15% DMSO	9	3	1
15% DMSO	13	13	1
**Total**	36	22	3

Table S1 above shows counting data of cells exposed to CPA mixtures. A clear cell response was when a cell shrunk and expanded rapidly without a clear separation of the cell membrane from the cell wall (plasmolysis). As these data show, a plasmolysis/deplasmolysis cycle was unusual even when most visible cells did respond.

CARS Images

The following Figures S6-S9 all show CARS data of rice callus cells after at least 3 minutes of d_6-DMSO exposure. Exposure times changed as the time to prepare the sample, find healthy cells, and begin imaging changed. All the cells shown in these figures exhibit apparent sequestering of d_6-DMSO within organelles inside the cells.

The left side of Figure S5 shows some callus with a particularly large cell towards the upper left. This cell is comparable in size to the one indicated in Figure S3, and the deplasmolysis of this cell was captured in the time-lapse images, though only one image is shown in the figure. The right side of Figure S5 shows the autofluorescence image captured simultaneously with the CARS images. Looking at the autofluorescence images, it is clear that organelles are moving around within the cells, indicated the cells are likely alive.

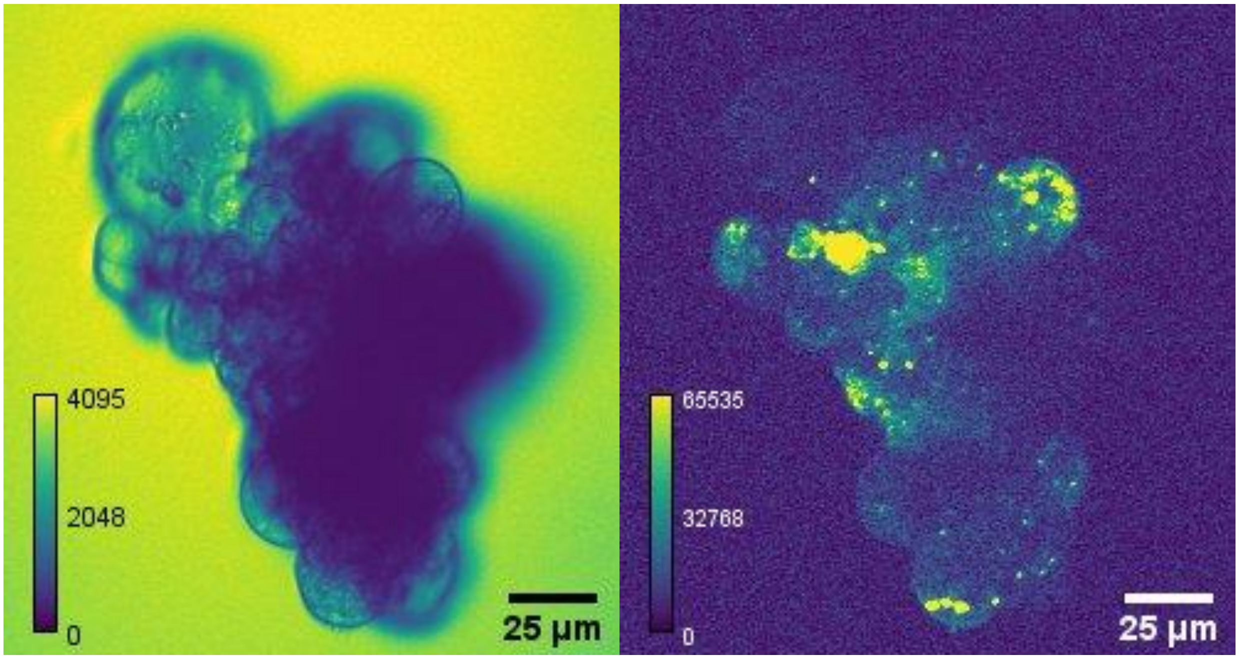

Figure S5. (LEFT) Rice callus after 5 minutes of 15% d_6-DMSO in distilled water exposure imaged with CARS microscope. Yellow represents higher d_6-DMSO signal, thus higher d_6-DMSO concentration. (RIGHT) Autofluorescence image taken simultaneously with the CARS image. Images were artificially colored with ImageJ LUT, mpl-viridis (26).

Figure S6 shows a cluster of callus cells after 8 minutes of exposure to a mixture of 15% d_6-DMSO in a sugar solution. In this figure there appears to be differences in d_6-DMSO across the cells, interestingly with apparent lack of d_6-DMSO inside come cell cavities and higher concentration, again, in circular organelles in the cells. This figure demonstrates that non-uniformity in the cellular components may lead to increased non-uniformity of the cryoprotectant distribution within the cells.

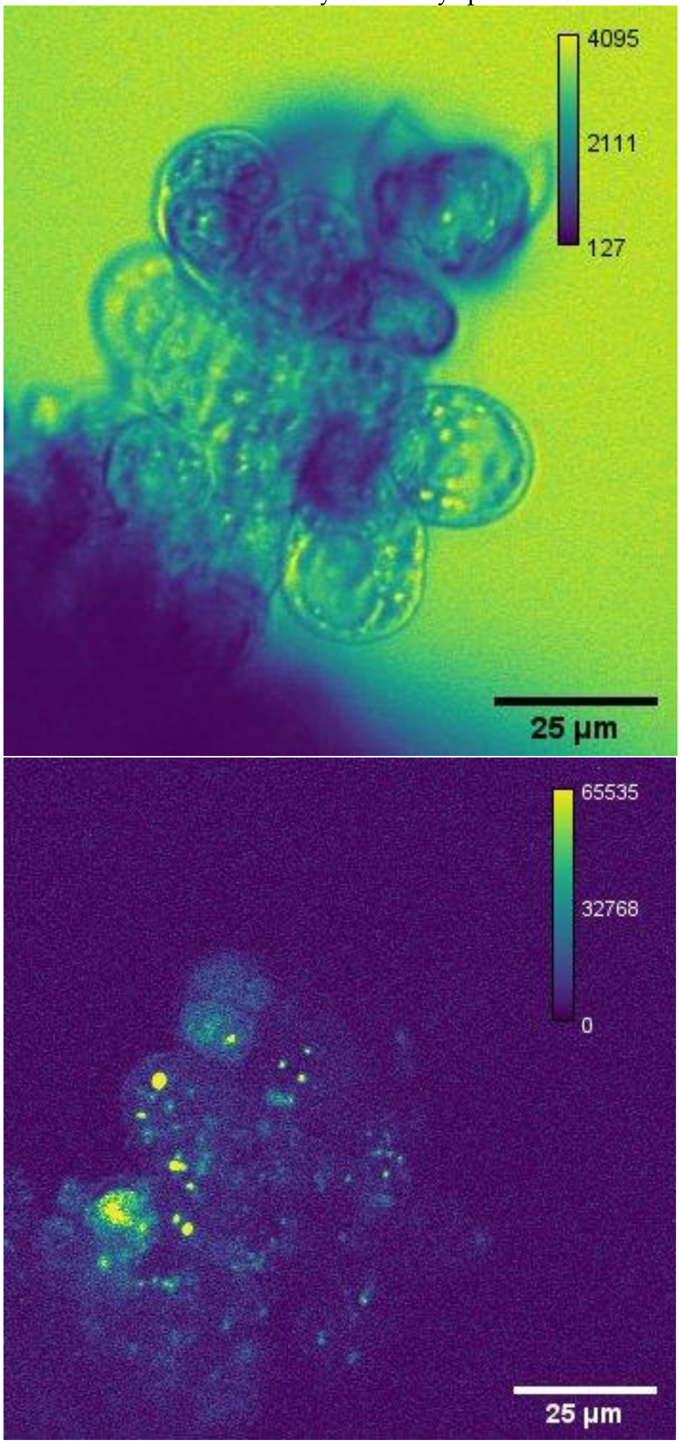

Figure S6. (TOP) Rice cells after 8 minutes of 15% d_6-DMSO in sugar water exposure imaged with CARS microscope. Yellow represents higher d_6-DMSO signal, thus higher d_6-DMSO concentration. (BOTTOM)

Autofluorescence image taken simultaneously as CARS data. When viewed in time-lapse, it is evident that the cells have internal movement, indicating that they are alive. Images were artificially colored with ImageJ LUT, mpl-viridis (26).

Figure S7 shows a CARS image of rice callus cells after 10 minutes of d_6-DMSO exposure. This figure has a range of cells in that some of them appear almost empty while others clearly contain organelles that have concentrated d_6-DMSO within them. It appears as though cells that lack organelles do have fairly uniform d_6-DMSO distribution while those cells that are densely packed with organelles have d_6-DMSO concentrating within the organelles. This contrast in cellular components and consequentially d_6-DMSO distributions within the cells further illustrates the necessity of considering that the organelle content of the cells likely will affect uniform cryoprotectant distribution within those cells. The lower image in Figure S7 shows the autofluorescence captured simultaneously with the CARS data.

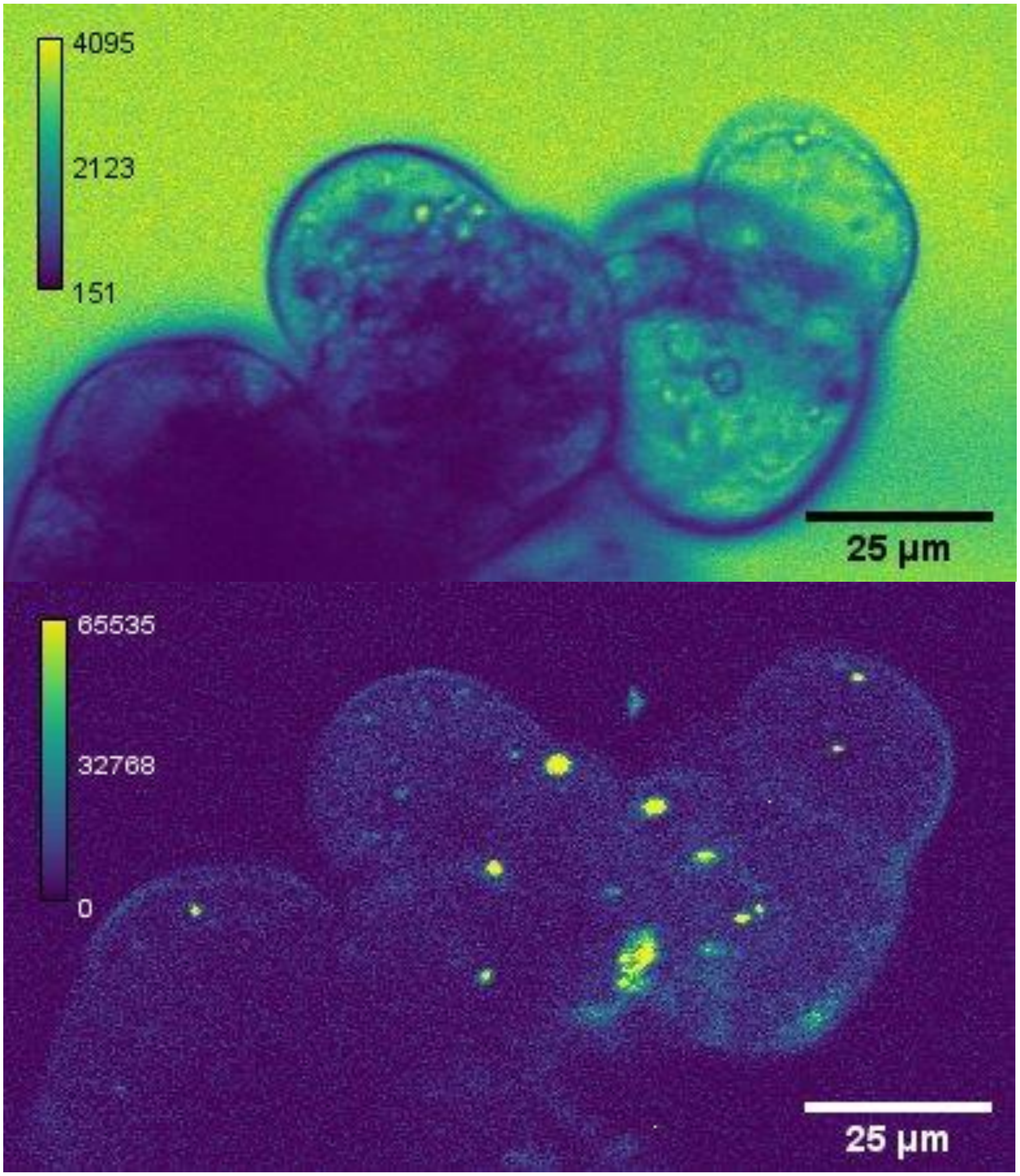

Figure S7. (TOP) Rice cells after 10 minutes of 15% d_6-DMSO in distilled water exposure imaged with CARS microscope. Yellow represents higher d_6-DMSO signal, thus higher d_6-DMSO concentration. (BOTTOM) Autofluorescence captured simultaneously with the CARS data showing an image of autofluorescent components of the cells. When viewed as a time-lapse, it is clear that components of the cell are moving around, indicating the cells are likely alive. Images were artificially colored with ImageJ LUT, mpl-viridis (26).

Figure S8 is the same Figure 2 in the primary document, with the addition of the pixel gray-scale value bar. This bar shows how different the gray-scale values are for the organelles contained within the outer visible cells of the callus and those in the center of the callus. The center of the callus has the lowest values not because there is not d_6-DMSO penetrating into it, but rather because the callus is too dense for the d_6-DMSO to make it to the detector. The image below the CARS data shows the autofluorescence

image of the cells captured at the same time as the CARS image. When viewed as a time lapse, it is clear that the autofluorescent parts of the cells are moving rapidly around, indicating that the cells are alive.

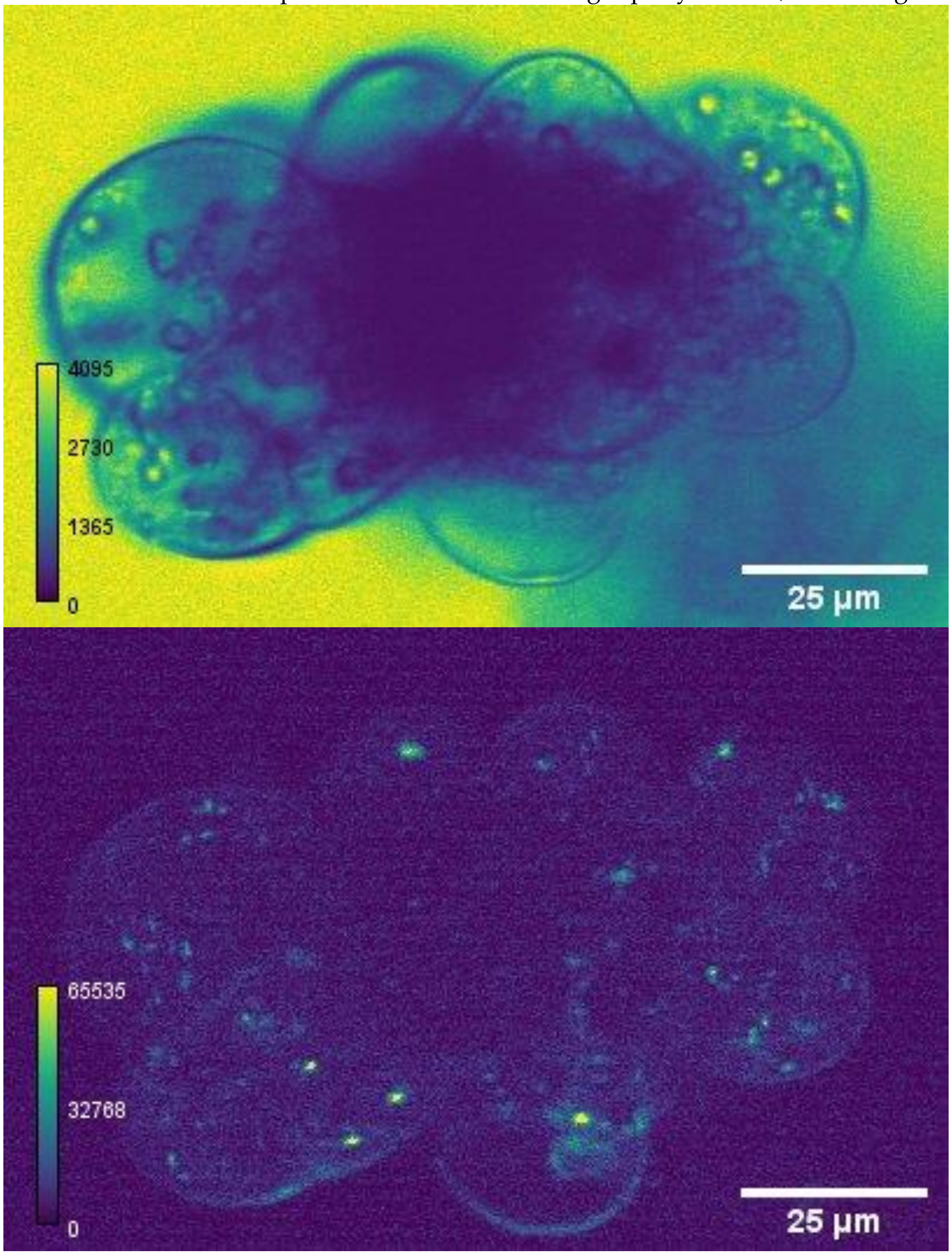

Figure S8. (TOP) Rice callus after 3 minutes of 15% d_6-DMSO in distilled water exposure imaged with CARS microscope. Yellow represents higher d_6-DMSO signal, thus higher d_6-DMSO concentration. (BOTTOM) Shows autofluorescence of the cells simultaneously collected with the CARS data. When viewing the time lapse data, the autofluorescencent components move around rapidly indicating that the cells of interest are alive. Images were artificially colored with ImageJ LUT, mpl-viridis (26).

Figures S9-11 further demonstrate that the pixel intensities within these organelles tend to be higher than the surrounding cytoplasm and, in some cases, as high as the d_6-DMSO solution surrounding the calli. This indicates that d_6-DMSO is likely sequestering within these organelles, not uniformly

distributed throughout the cell. In all cases, the trace starts at the number outside the cell and moves into the cell.

In Figure S9, two organelles are bisected by Line 1 and Line 3. When compared to the accompanying background traces (Line 2 and Line 4 respectively) the intensities in these organelles are much higher. In both Line 1 and Line 3, the intensity of the pixels within the organelle reach that of those outside the cell, suggesting that the d_6-DMSO concentration within the organelle is at least 15%, as that is the d_6-DMSO concentration in the surrounding solution.

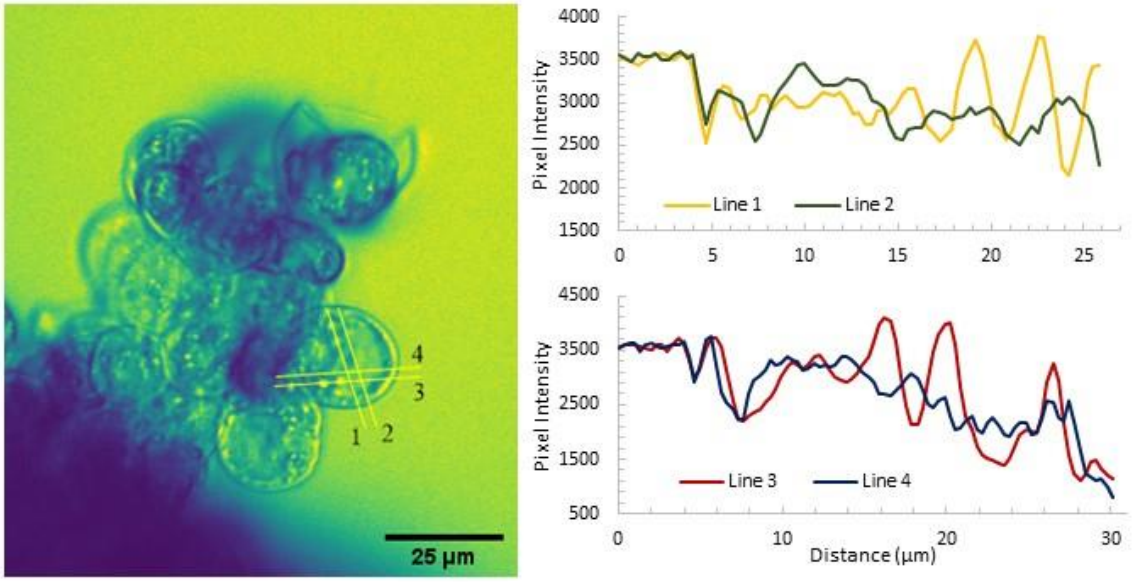

Figure S9. CARS image shown in Figure S6 with four traces analyzed for pixel intensity. Line 2 is a background trace for Line 1, and Line 4 is a background trace for Line 3.

Figure S10 shows five traces over three different cells. Lines 1-3 show various intensities within one cell. When compared to each other, it is clear that when the trace bisects an organelle, there is a distinct shape in the pixel intensity – dipping to low intensity before rapidly rising, and then dipping again. Additionally, the pixel intensity decreases rapidly when the trace goes over the cell wall. This indicates that the d_6-DMSO is likely passing through the cell wall and membranes without concentrating within those structures.

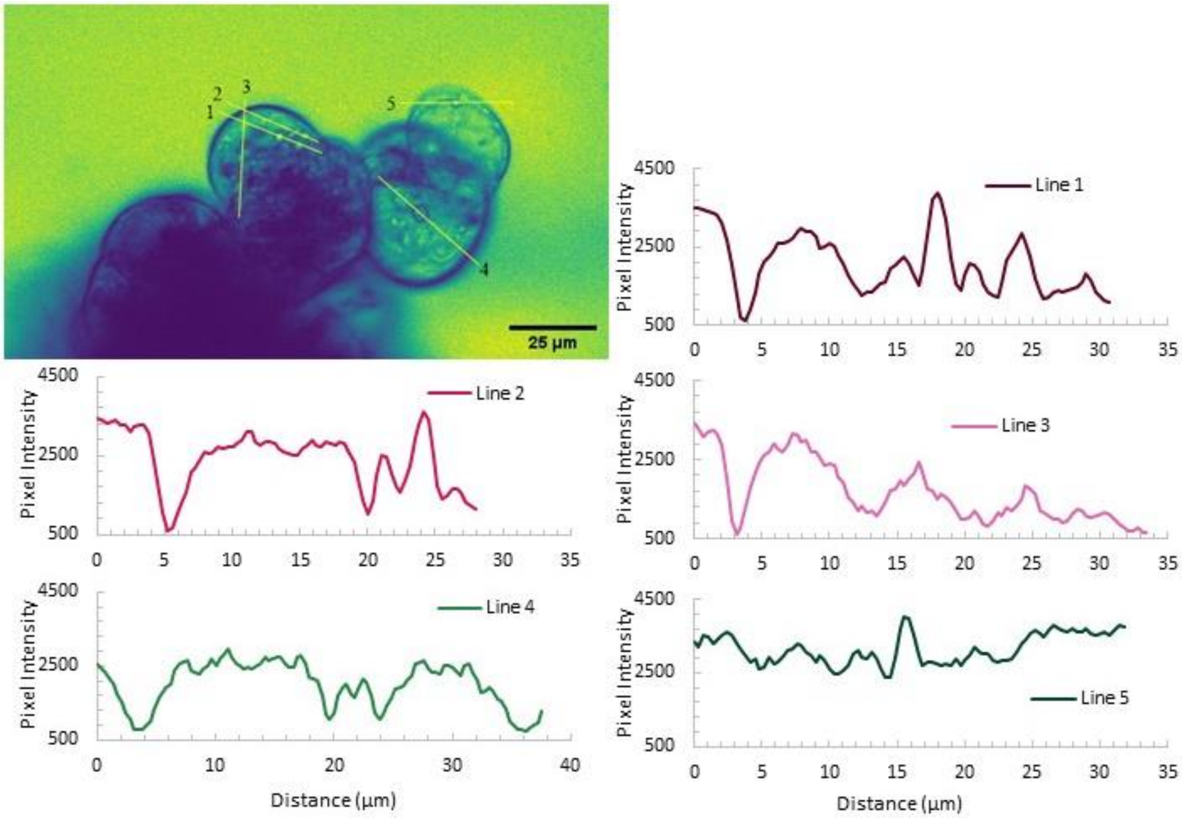

Figure S10. CARS image shown in Figure S7 with five traces analyzed for pixel intensity. All traces are independent of each other; there are no background traces.

Finally, Figure S11 shows other cells within the cluster shown in Figure 2 in the manuscript. These cells are not as clear as the one shown in Figure 2, but still present the unique line shape when the trace bisects an organelle.

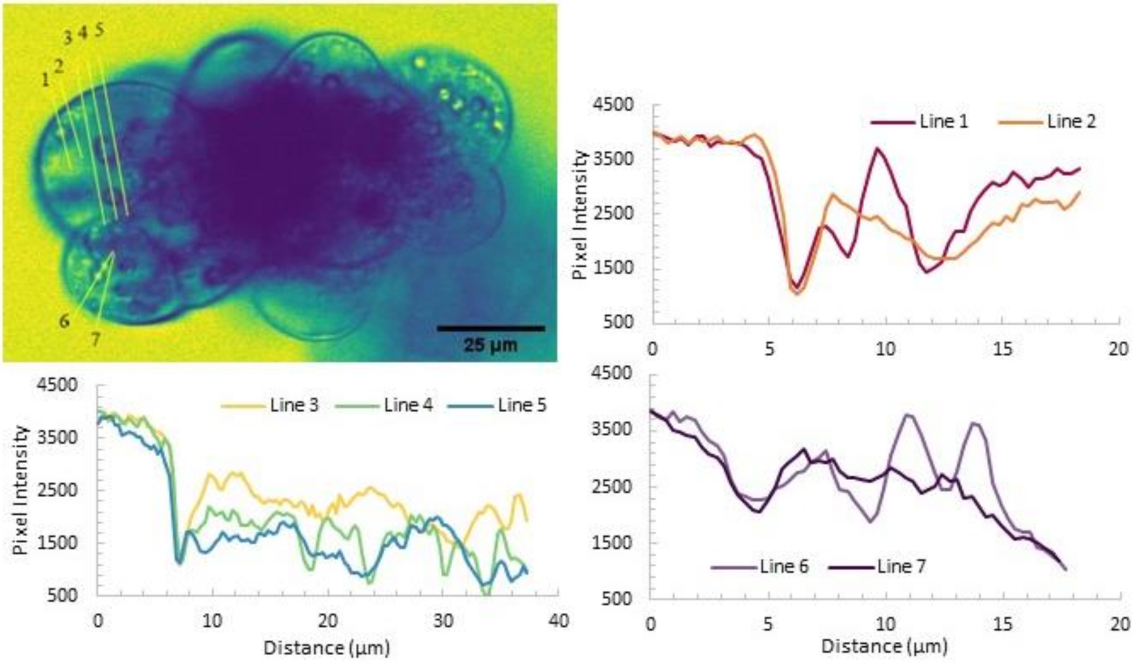

Figure S11. CARS image shown in Figure 2 (manuscript) and Figure S8 showing seven additional traces analyzed for pixel intensity. Line 2 is a background for Line 1, Lines 3 and 5 sandwich Line 5 to demonstrate the difference in the surrounding cell on either side of those bisected organelles, and Line 7 is a background for Line 6.
